# Changes in serum zonulin in individuals with morbid obesity after weight-loss interventions: a prospective cohort study

**DOI:** 10.1186/s12902-020-00594-5

**Published:** 2020-07-22

**Authors:** Martin Aasbrenn, Stian Lydersen, Per G Farup

**Affiliations:** 1 Department of Surgery, Innlandet Hospital Trust, Gjøvik, Norway; 2 Novo Nordisk Foundation Center for Basic Metabolic Research, University of Copenhagen, Copenhagen, Denmark; 3 Regional Centre for Child and Youth Mental Health and Child Welfare, Trondheim, Norway; 4 Department of Research, Innlandet Hospital Trust, Brumunddal, Norway

**Keywords:** Obesity, Intestinal permeability, Zonulin, Bariatric surgery, Diet interventions

## Abstract

**Background:**

Zonulin is a biomarker of impaired intestinal permeability, which has been associated with various disorders. The primary aim was to study serum zonulin (s-zonulin) in individuals with morbid obesity before and after a conservative weight loss intervention followed by bariatric surgery. The secondary aims were to explore predictors of s-zonulin, and the associations between the changes of the predictors and changes in s-zonulin, and to compare the associations in the two treatment periods.

**Methods:**

Individuals with morbid obesity were included. Data before any weight loss interventions, after a 6 months’ conservative weight loss intervention, and 6 months after bariatric surgery were used. S-zonulin was measured with an ELISA method from Immundiagnostik AB, Bensheim, Germany. Data were analysed with mixed models.

**Results:**

The mean body mass index was 42.1 kg/m^2^ (SD 3.8) at inclusion and was reduced to 38.7 kg/m^2^ (SD 3.8) and 29.8 kg/m^2^ (SD 3.8) after the conservative treatment and bariatric surgery respectively. S-zonulin was 63 ng/mL (SD 32) at inclusion and was reduced with 19 ng/ml (95% CI 12 to 26, *p* < 0.001) after conservative treatment and 11 ng/ml (95% CI 0 to 21, *p* = 0.04) after bariatric surgery. At inclusion, s-zonulin was significantly associated with factors including p-glucose (B = 2.21, 95% CI 1.09 to 3.33, *p* < 0.001), c-reactive protein (B = 1.02, 95% CI 0.45 to 1.58, *p* < 0.001) and the intake of proteins (B = 0.23, 95% CI 0.08 to 0.38, *p* = 0.003) and non-nutritive sweeteners (B = 0.68, 95% CI 0.19 to 1.17, *p* = 0.007). The reduction in s-zonulin after the conservative weight loss intervention was significantly associated with improvement in diarrhoea (B = 6.6, 95% CI  1.3 to 11.8, *p* = 0.02), HbA1c (B = 9.7, 95% CI 1.1 to 18.3, *p* = 0.03), p-glucose (B = 3.5, 95% CI 1.2 to 5.9, *p* = 0.004) and gamma-GT (B = 0.28, 95% CI 0.09 to 0.47, *p* = 0.004), but not associated with the change in body mass index (B = 0.9, 95% CI − 1.5 to 3.3, *p* = 0.46).

**Conclusions:**

S-zonulin was markedly reduced after the conservative weight loss intervention, and further reduced after bariatric surgery. The reduction in s-zonulin was associated with improvement of diarrhoea, markers of glucose intolerance and liver disease, but not associated with the change in body mass index.

## Background

Intestinal permeability is increased in individuals with obesity. Zonulin, a 47 kDa protein, is involved in the regulation of the paracellular permeability in the intestine [1, 2]. The protein is secreted by the liver, the intestine and several other tissues, circulates in the blood and binds to receptors on the enterocytes in the ileum and jejunum [1, 3]. Binding to these receptors leads to reversible modulation of intercellular tight junctions and thereby increased small intestinal paracellular permeability [1, 2]. Zonulin may also impact the blood-brain barrier through similar mechanisms [4]. Zonulin can be measured in the blood and the faeces. High serum zonulin (s-zonulin) has been interpreted as increased intestinal permeability [1, 2, 5]. Measured with the available enzyme-linked immunosorbent assay (ELISA) methods, s-zonulin has been associated with obesity and high energy intake in cross-sectional studies [3, 6–9]. S-zonulin has also been associated with the comorbidities of obesity, such as fatty liver disease and diabetes mellitus [6, 10].

An altered intestinal paracellular permeability may be a mediator between the intestinal environment and metabolic disorders [11]. Zonulin inhibitors can reduce the paracellular permeability, and may become useful as drugs in the future [12]. Studies that go beyond cross-sectional examinations, such as longitudinal studies and intervention studies are needed to explore such relations.

A few longitudinal studies of zonulin before and after conservative weight-loss interventions have been performed, but no studies of the changes after bariatric surgery [5, 13, 14]. If the changes in s-zonulin partly explain the metabolic changes after bariatric surgery, zonulin inhibitors could have an effect on some of the metabolic disorders related to obesity.

The primary aim was to study s-zonulin in individuals with morbid obesity before and after a conservative weight loss intervention followed by bariatric surgery. The secondary aims were to explore predictors of s-zonulin, and the associations between the changes of the predictors and changes in s-zonulin, and to compare the associations in the two treatment periods.

## Methods

### Study design and setting

All adult individuals referred to an outpatient obesity clinic at Innlandet Hospital Trust in Gjøvik, South-Eastern Norway from December 2012 to September 2014 were invited to participate in a prospective cohort study. The three first visits of the prospective study were used in this substudy; the first visit was before any weight loss interventions, the second visit was after a 6 month conservative weight loss intervention and before bariatric surgery, and the third visit was 6 months after bariatric surgery.

### Participants

The inclusion criteria were individuals 18–65 years of age with morbid obesity, defined as body mass index (BMI) above 40 kg/m^2^ or BMI above 35 kg/m^2^ with comorbidities related to obesity [15]. Exclusion criteria were previous major gastrointestinal surgery, organic gastrointestinal disorders, alcohol and drug addiction, major psychiatric disorders, and serious somatic disorders not related to obesity.

### Interventions

The included patients did all follow routine clinical care at the obesity center, no interventions were altered because patients were included in the cohort study.

#### The conservative weight loss intervention

The conservative weight loss intervention with group counselling and personalised lifestyle interventions was based on the weight loss programs used in most Norwegian obesity clinics in this period of time [16], with some small local changes from the national standard due to availability of personnel. The intervention was organised as a series of outpatient visits [16, 17]. The intervention started with three outpatient consultations of one-hour duration with a nurse, a nutritionist and a physician. These three consultations included registration of the patient’s current diet and physical activity and personalised advice about adjustments. A few weeks later, the participants were enrolled in groups with meetings of 4 h duration during seven consecutive weeks. These seven meetings were based on group counselling and were led by nurses specialised in obesity, surgeons, nutritionists, and a psychologist.

The dietary recommendations were based on a reduced daily energy intake and recommendation of food items with a high micronutrient content [17]. The participants were adviced to eat less sugar and fat and more fibre and protein. They were recommended to distribute their food intake into 4–6 daily meals with 2–4 h intervals. The nutritionists gave personalised advice on specific dishes based on what the participants liked to eat and what they were used to eat. At a time point 21 days before the second study visit, the participants were recommended to change to a more standardised “crisp bread diet” with 4200 kJ daily energy intake. During the crisp bread diet, the participants were recommended to eat six units of crisp bread with high-protein, low-fat topping (as fish, cheese or meat) and 0.45 L of milk. They were adviced to eat dishes based on meat or fish with vegetables on small plates for dinner. Participants who preferred meal replacement powder to the crisp bread diet could use meal replacement powder giving 3765 kJ with free amounts of vegetables these 21 days. Concerning the choice of vegetables, corn, olives or avocados were not recommended in this period due to the energy content. All kinds of beverages without energy content were allowed during the conservative weight loss intervention.

#### Bariatric surgery

Bariatric surgery was performed as Roux-en-Y Gastric Bypass or Gastric Sleeve. Three experienced surgeons performed all procedures in a standardised manner with a laparoscopic approach [18, 19].

### Study visits

#### The first visit

At the first visit, BMI and waist-hip-ratio were measured and the participant filled in a case report form with assistance from a study nurse with questions about demographics, diseases and health scores. Blood samples were drawn. The participants were asked to bring along a faecal sample and a filled-in food frequency questionnaire (FFQ) at the next visit.

#### The second visit

The second study visit was scheduled after the conservative weight loss intervention before bariatric surgery. BMI was measured, the participants filled in a case report form, and blood samples were drawn. A faecal test and the FFQ were not included at this visit, however, all participants used a standardised diet at this time point.

#### The third visit

The third study visit was scheduled 6 months after bariatric surgery. BMI was measured, a case report form was filled out, blood samples were drawn and the participant was asked to bring along a faecal sample test and a filled-in FFQ.

### Variables

#### Anthropometrics

Weight and height were measured to calculate BMI (kg/m^2^) at all three visits. The waist circumference was measured at the smallest part of the waist, the hip circumference was measured at the widest parts of the buttocks, and waist-hip-ratio was calculated.

#### Demographics

Age (years), sex (male/female) and smoking habits (daily smoking/no daily smoking) were registered.

#### Diseases

Irritable bowel syndrome was diagnosed with the Rome III criteria [20].

#### Health scores

The participants reported gastrointestinal symptoms on the gastrointestinal symptom rating scale for patients with irritable bowel syndrome (GSRS-IBS). These responses were used to register values for constipation and diarrhoea according to two predefined subscales, GSRS-constipation and GSRS-diarrhoea [21]. These two scales range from 1 to 7, high values indicate many gastrointestinal symptoms.

#### Blood tests

Serum and EDTA plasma were immediately analysed or stored at − 80 °C for later analysis. C-reactive protein (CRP) and HbA1c were analysed at Innlandet Hospital Trust Gjøvik with a Cobas c501 instrument with the reagents CRPL3 and Tina-quant HbA1C (Roche Diagnostics GmbH, Mannheim, Germany). IL-6 was analysed with Immulite 2000, TNF alpha was analysed with Immulite 1000 (Siemens Healthcare AS, Oslo, Norway). Plasma glucose (p-glucose) and gamma-glutamyl transferase (gamma-GT) were analysed on Cobas 6000, with instruments and reagents delivered by Roche Diagnostics Norway. S-zonulin was analysed by a commercially available ELISA kit (Immundiagnostik, Germany, normal values < 38 ng/ml.)

#### Faecal tests

The faecal microbiota was assessed with the GA-map dysbiosis test manufactured and marketed by Genetic Analysis, Oslo, Norway. This CE-marked test was created based on selected 16S rRNA gene sequences from bacteria: Probes were selected based on their ability to separate individuals with disease from healthy individuals. Details about the creation process and the test are given in a publication from the manufacturer [22]. The results were given from the manufacturer as a score on a dysbiosis index ranging from 1 to 5, where values above 2 are defined as dysbiosis [22].

#### Dietary intake

The intake of energy, proteins, fats, carbohydrates, fibres, starch and sugar was registered with a semiquantitative food frequency questionnaire designed for the Norwegian population [23]. The intake of non-nutritive sweeteners (NNS) was estimated from the same questionnaire, one unit was defined as the quantity that corresponds to 100 ml of beverage with NNS, or two tablets for use in coffee or tea [24].

### Statistics

The characteristics of all participants at three visits are presented as means with standard deviation or ratio with percentage. To answer the primary aim; the changes in s-zonulin after the two weight-loss interventions, we used a linear mixed model with s-zonulin as the dependent variable, patient as random effect and time point as a three level categorical covariate. To answer the first part of the secondary aim concerning the predictors of s-zonulin, we added potential explanatory variables one at a time as covariates in the mixed model, including the interaction between the potential explanatory variable and the time point. S-zonulin was dependent variable and patient was random effect. This analysis was done for the whole treatment period and for the three visits separately. To answer the second part of the secondary aim, the associations between the changes of the predictors and changes in s-zonulin, we used mixed models with the change of s-zonulin as dependent variable, patient as random effect and treatment period as a two level categorical covariate. Subsequently, we added changes in the potential explanatory variables one at a time as covariates in the mixed model, including the interaction between the change in the potential explanatory variable and the treatment period. Sex, age and BMI were included as covariates in all mixed model analyses. Normality of residuals was checked by visual inspection of Q-Q plots. Due to multiple hypotheses, *p*-values between 1 and 5% should be interpreted with caution. A post-hoc exploration of variables independently associated with s-zonulin was performed with backwards elimination of covariates with *p* > 0.05 from a model with all relevant explanatory covariates. The analyses were performed with SPSS 25.

### Sample size

The sample size was fixed because the cohort was from a previously performed study and a power calculation was not performed [25].

### Ethical approval

The study was approved by the Regional Committees for Medical and Health Research Ethics South East Norway (reference 2012/966) and conducted in accordance with the Declaration of Helsinki including written informed consent from all the participants.

## Results

**Fig. 1:**
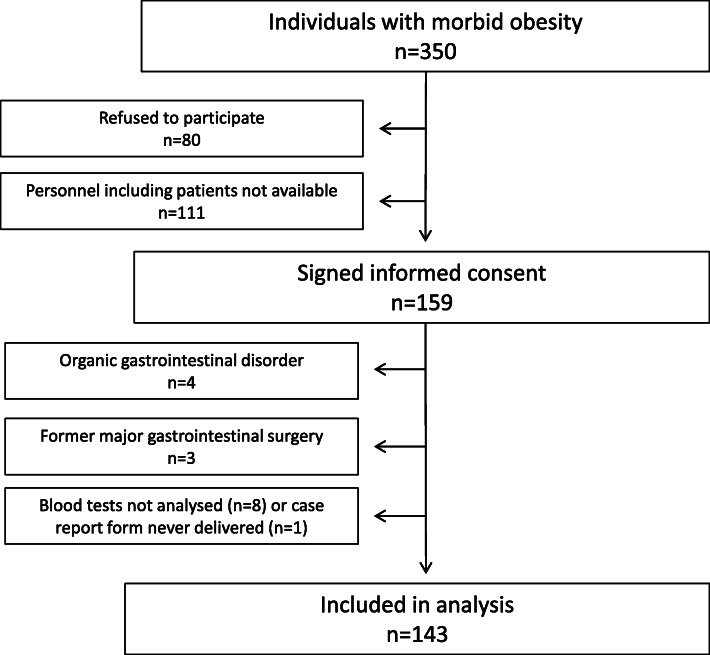
Inclusion of participants

In all, 159 individuals gave informed consent, 16 individuals were later excluded due to organic disease or missing information. Figure 1 shows a flow chart of the individuals. Data from 143 individuals (77% women) with a mean age of 43.0 years (standard deviation (SD) 8.7) and BMI of 42.1 kg/m^2^ (SD 3.8) was included in the analyses. Table 1 gives the participants’ characteristics. BMI was reduced to 38.7 kg/m^2^ (SD 3.8) and 29.8 kg/m^2^ (SD 3.8) after the conservative treatment and bariatric surgery, respectively. This corresponded to a mean total weight loss of 36.8 kg (SD 9.4) in the period as a whole.

**Table 1:** Participant characteristics before and after the conservative and surgical weight loss interventions

	At inclusion		After the conservative weight loss intervention		After bariatric surgery	
	n		N		n	
Sex (%Female)	143	110 (77%)				
Age (years)	143	43.0 (8.7)				
Current smoker	138	25 (18%)				
Weight (kg)	140	123.5 (18.8)	108	114.0 (17.8)	73	87.5 (15.4)
Body mass index (kg/m^2^)	140	42.1 (3.8)	107	38.7 (3.8)	72	29.8 (3.8)
Waist-hip-ratio	131	0.98 (0.10)	8	0.98 (0.14)	26	0.94 (0.09)
Irritable bowel syndrome (yes/no)	133	36 (27%)	91	18 (20%)	76	20 (26%)
GSRS-constipation	134	1.5 (0.8)	95	1.6 (0.9)	73	1.8 (0.9)
GSRS-diarrhoea	127	1.8 (1.0)	89	1.5 (0.9)	80	1.6 (0.9)
HbA1c (%)	143	5.9 (1.4)	99	5.5 (0.9)	96	5.2 (0.8)
Plasma glucose (mmol/L)	143	6.6 (3.1)	110	5.8 (1.7)	96	5.4 (1.2)
S-Zonulin (ng/ml)	141	63 (32)	96	43 (7)	94	29 (18)
C-reactive protein (mg/L)	143	6.9 (6.2)	110	4.4 (3.9)	96	1.9 (2.5)
IL-6 (ng/mL)	139	2.8 (2.9)		NA	94	2.2 (1.4)
TNF (ng/mL)	140	12.1 (41.9)		NA	94	10.6 (38.1)
Gamma-GT (U/L)	143	43 (45)	98	32 (24)	96	17 (13)
Nutritional intake of						
Energy (MJ)	96	11 (4)		NA	71	6 (5)
Proteins (g)	96	111 (35)		NA	71	75 (58)
Fats (g)	96	99 (46)		NA	71	58 (49)
Carbohydrates (g)	96	275 (125)		NA	71	158 (110)
Starch (g)	96	134 (50)		NA	71	69 (38)
Fibre (g)	96	34 (10)		NA	71	22 (10)
Sugar (g)	96	47 (75)		NA	71	19 (26)
Non-nutritive sweeteners (units^a^)	96	8 (11)		NA	71	6 (7)
Dysbiosis Index^b^	100	2.8 (1.3)		NA	42	4.1 (1.0)

Data are given as mean (standard deviation) or number (percentage)

*S-zonulin* Serum-zonulin, *GSRS* Gastrointestinal symptom rating scale, *TNF* Tumor necrosis factor, *Gamma-GT* Gamma-glutamyl transferase, *NA* Not available

^a^One unit corresponds to 100 ml of beverage with non-nutritive sweeteners

^b^The dysbiosis index range from 1 to 5, values > 2 indicate gut microbial dysbiosis

The mean s-zonulin at inclusion was 63 ng/ml (SD 32). The value was reduced with 19 ng/ml (95% confidence interval (CI) 12 to 26) after the conservative weight loss intervention and further reduced with 11 ng/ml (95% CI 0 to 21) after the bariatric surgery (Table 2).

**Table 2:** Trajectory of s-zonulin before and after the conservative and surgical weight loss interventions

	Estimate (95% confidence interval of the mean)	*P*-values
At inclusion (ng/ml)	62 (57 to 66)	**< 0.001**
Change from baseline to after the surgical intervention (during both interventions) (ng/ml)	−29 (− 17 to −41)	**< 0.001**
Change during the conservative weight loss intervention (ng/ml)	−19 (− 12 to −26)	**< 0.001**
Change during the surgical weight loss intervention (ng/ml)	− 11 (0 to −21)	**0.04**

Analysis with linear mixed models, with zonulin as the dependent variable, patient as random effect and the covariates time, sex, age (centred at age 43 years) and body mass index (measured at three time points, centred at body mass index 39 kg/m^2^) as fixed effects

*S-zonulin* Serum zonulin

At inclusion, high s-zonulin values were associated with a high waist-hip-ratio, irritable bowel syndrome and diarrhoea, and with high values of HbA1c, p-glucose, CRP, gamma-GT and the dysbiosis index. High s-zonulin values were also associated with a high dietary intake of energy, proteins, fats and NNS. S-zonulin was not associated with BMI. At the second and third study visit, s-zonulin was not significantly associated with any of the explanatory variables. Table 3 shows both the overall associations between s-zonulin and the explanatory variables and the associations at each of the three points of time. Table 4 shows associations between changes in s-zonulin and the changes in the explanatory variables. The reduction in s-zonulin after the conservative weight loss intervention was associated with reductions in GSRS-diarrhoea, HbA1c, p-glucose and gamma-GT. No significant associations between the reductions in s-zonulin and the explanatory variables were observed after the surgical weight loss intervention. The difference between the associations after the conservative and after the surgical period was statistically significant for GSRS-diarrhoea, HbA1c and p-glucose.

**Table 3:** Associations between s-zonulin and the explanatory variables overall and at the three points of time

	Overall		At inclusion		After the conservative weight loss intervention		After bariatric surgery	
	B (95% CI)	*p*	B (95% CI)	*p*	B (95% CI)	*p*	B (95% CI)	*p*
Sex (Female)	−1.32 (− 8.05 to 5.40)	0.70						
Age	0.19 (− 0.15 to 0.54)	0.28						
Body mass index	0.39 (−0.40 to 1.18)	0.33						
Waist-hip-ratio	84.2 (18.2 to 150.2)	**0.013**	110.1 (39.1 to 181.1)	**0.003**	3.4 (− 154.7 to 161.5)	0.97	9.1 (− 122.7 to 140.9)	0.89
Irritable bowel syndrome	5.82 (−1.08 to 12.72)	0.10	10.16 (0.70 to 19.61)	**0.04**	1.19 (−11.80 to 14.17)	0.86	1.20 (−12.95 to 15.34)	0.87
GSRS-constipation	−0.23 (−3.56 to 3.09)	0.89	− 0.05 (−5.17 to 5.06)	0.98	− 0.01 (− 5.54 to 5.53)	1.00	− 0.91 (−7.75 to 5.93)	0.79
GSRS-diarrhoea	3.00 (0.06 to 5.95)	**0.046**	5.36 (1.30 to 9.42)	**0.01**	0.49 (−5.45 to 6.43)	0.87	0.42 (−5.50 to 6.33)	0.89
HbA1c (%)	4.44 (1.92 to 6.96)	**0.001**	5.48 (2.54 to 8.42)	**< 0.001**	1.39 (−4.01 to 6.78)	0.61	2.75 (−3.75 to 9.25)	0.41
Plasma glucose (mmol/L)	2.21 (1.09 to 3.33)	**< 0.001**	2.56 (1.30 to 3.81)	**< 0.001**	0.72 (−2.01 to 3.45)	0.60	1.58 (−2.44 to 5.59)	0.44
C-reactive protein (mg/L)	1.02 (0.45 to 1.58)	**< 0.001**	1.17 (0.52 to 1.82)	**< 0.001**	0.64 (−0.56 to 1.85)	0.30	0.37 (−1.67 to 2.42)	0.72
IL-6 (ng/mL)	1.51 (−0.04 to 3.07)	0.06	1.28 (−0.39 to 2.95)	0.13	NA		3.11 (−1.26 to 7.48)	0.16
TNF (ng/mL)	0.02 (−0.07 to 0.11)	0.69	0.03 (−0.08 to 0.14)	0.59	NA		−0.01 (− 0.16 to 0.15)	0.95
Gamma-GT (U/L)	0.16 (0.08 to 0.24)	**< 0.001**	0.18 (0.09 to 0.27)	**< 0.001**	0.02 (−0.18 to 0.22)	0.85	0.14 (−0.29 to 0.58)	0.52
Nutritional intake of								
Energy (kJ)	1.87 (0.43 to 3.31)	**0.01**	2.30 (−0.71 to 3.90)	**0.005**	NA		−0.16 (4.05 to 3.73)	0.94
Proteins (g)	0.23 (0.08 to 0.38)	**0.003**	0.28 (0.11 to 0.44)	**0.001**	NA		0.007 (−0.325 to 0.339)	0.97
Fats (g)	0.18 (0.05 to 0.30)	**0.006**	0.19 (0.06 to 0.32)	**0.003**	NA		−0.06 (−0.47 to 0.35)	0.77
Carbohydrates (g)	0.03 (−0.01 to 0.08)	0.13	0.04 (−0.01 to 0.08)	0.12	NA		0.004 (−0.125 to 0.134)	0.95
Starch (g)	0.54 (−0.59 to 0.17)	0.35	0.07 (−0.05 to 0.19)	0.26	NA		−0.04 (− 0.32 to 0.24)	0.78
Fibre (g)	0.34 (−0.17 to 0.84)	0.19	0.50 (−0.07 to 1.08)	0.09	NA		−0.27 (−1.23 to 0.70)	0.59
Sugar (g)	0.03 (−0.05 to 0.11)	0.46	0.03 (−0.05 to 0.11)	0.48	NA		0.10 (−0.46 to 0.67)	0.72
Non-nutritive sweeteners (u)^a^	0.68 (0.19 to 1.17)	**0.007**	0.76 (0.23 to 1.30)	**0.006**	NA		0.30 (−0.72 to 1.32)	0.56
Dysbiosis index^b^	6.95 (3.11 to 10.80)	**0.001**	6.76 (2.32 to 11.19)	**0.003**	NA		7.54 (−1.35 to 16.43)	0.10

Analyses with linear mixed models with zonulin as dependent variable, and patient as random effect. The fixed effects in the left column are sex, age, body mass index, the explanatory variable in the same row and time (as a three level categorical covariate). In the three other columns, the fixed effects are sex, age, body mass index and time (with the time point given in the heading of the column as reference), the explanatory variable in the same row and the interaction term between this explanatory variable and time

*S-zonulin* Serum-zonulin, *TNF* Tumour necrosis factor, *GSRS* Gastrointestinal symptom rating scale, *NA* Not available, *Gamma-GT* Gamma-glutamyltransferase

^a^One unit corresponds to 100 ml of beverage with non-nutritive sweeteners

^b^The dysbiosis index range from 1 to 5, values > 2 indicate gut microbial dysbiosis

**Table 4:** Associations between the changes in s-zonulin and the changes in the factors of interest after the two weight-loss interventions

Change in covariate	Overall change		Change after conservative weight loss intervention		Change after bariatric surgery		Interaction	
	B (95% CI)	*p*-values	B (95% CI)	*p*-values	B (95% CI)	*p*-values	B (95% CI)	*p*-values
Body mass index (kg/m^2^)	0.9 (− 1.5 to 3.3)	0.46	3.2 (−0.9 to 7.2)	0.27	−0.4 (− 3.4 to 2.6)	0.80	3.5 (− 1.5 to 8.6)	0.17
Weight (kg)	−0.7 (− 3.9 to 2.5)	0.68	1.1 (− 2.7 to 5.0)	0.56	− 0.4 (− 3.6 to 2.9)	0.83	−1.5 (− 3.2 to 0.2)	0.08
GSRS-constipation	1.2 (− 4.4 to 6.9)	0.67	7.0 (−1.6 to 15.6)	0.11	−3.6 (− 11.1 to 3.8)	0.34	−10.6 (− 22.1 to 0.94)	0.07
GSRS-diarrhoea	6.6 (1.3 to 11.8)	**0.02**	13.6 (6.2 to 20.9)	**< 0.001**	−0.4 (− 7.7 to 6.9)	0.91	14.0 (3.5 to 24.4)	**0.009**
HbA1c (%)	9.7 (1.1 to 18.3)	**0.03**	16.1 (5.9 to 26.3)	**0.002**	−6.0 (−20.9 to 8.9)	0.43	−22.1 (−4.2 to −40.0)	**0.016**
Plasma glucose (mmol/L)	3.5 (1.2 to 5.9)	**0.004**	5.1 (2.4 to 7.7)	**< 0.001**	−2.6 (−7.9 to 2.7)	0.34	−7.6 (−1.7 to 13.6)	**0.012**
C-reactive protein (mg/L)	0.8 (−0.5 to 2.1)	0.22	1.2 (−0.4 to 2.9)	0.15	0.2 (−1.9 to 2.3)	0.88	−1.1 (−3.7 to 1.6)	0.43
Gamma-GT (U/L)	0.28 (0.09 to 0.47)	**0.004**	0.36 (0.15 to 0.58)	**0.001**	−0.02 (− 0.40 to 0.37)	0.93	0.38 (− 0.06 to 0.82)	0.09

Analyses with linear mixed models with zonulin changes as dependent variable, and patient as random effect. The fixed effects are sex, age, body mass index, intervention period (with the intervention period given in the heading of the column as reference), the explanatory variable in the same row and the interaction between this explanatory variable and the intervention period. The interaction terms between the intervention period and the explanatory variable in the same line are also presented separately in the columns to the right with title “Interaction”

*TNF* Tumour necrosis factor, *GSRS* Gastrointestinal Symptom Rating Scale, *Gamma-GT* Gamma-glutamyl transferase

In the post-hoc analysis, four explanatory variables were independently associated with s-zonulin: P-glucose (B = 2.14, 95% CI 0.46 to 3.81, *p* = 0.013), CRP (B = 1.85, 95% CI 0.88 to 2.83, *p* < 0.001), intake of proteins (B = 0.28, 95% CI 0.14 to 0.42, *p* < 0.001) and intake of NNS (B = 0.50, 95% CI 0.06 to 0.94, *p* = 0.03).

## Discussion

S-zonulin was high in a group of individuals with morbid obesity referred to a hospital for obesity treatment. High s-zonulin at inclusion was associated with their diet, and with diarrhoea, dysbiosis and biomarkers of metabolic unhealth. S-zonulin was partly normalised after a six-month long conservative weight loss intervention and further reduced 6 months after bariatric surgery. The changes in s-zonulin after the weight-loss interventions were not significantly associated with the changes in BMI.

The mean s-zonulin at baseline was 63 ng/ml, higher than the upper limit of the reference interval. S-zonulin was reduced after both the weight-loss interventions. The change in s-zonulin was most prominent after the conservative weight loss intervention, before the substantial changes in BMI induced by surgery. A reduction in intestinal permeability after a conservative weight loss intervention has also been observed in a study where the permeability was measured with a dual sugar test in individuals with an average BMI around 44 kg/m^2^ [13]. On the contrary, an increase in s-zonulin after a dietary intervention was observed in a study where the participants had type 2 diabetes and mean BMI around 30 kg/m^2^ [14]. The current study indicates that both conservative and surgical weight loss interventions can lead to reduced s-zonulin in individuals with BMI 42 kg/m^2^. Differences between individuals with overweight and severe obesity or differences between the diets used in the different studies might explain the diverging findings.

Several explanatory variables were associated with s-zonulin at the first of the three visits. Abdominal obesity (waist-hip ratio), a marker of fatty liver disease (gamma-GT), reduced glucose tolerance (HbA1C) and increased low-grade inflammation were associated with high levels of s-zonulin, in accordance with earlier studies [3, 5, 6, 8, 26]. S-zonulin was not associated with BMI, but the levels of s-zonulin in the cohort as a whole were much higher than previously observed levels of s-zonulin in individuals without obesity [6, 8].

Gluten and bacteria in the intestinal lumen are among the triggers that can lead to zonulin secretion from the liver and intestine [1]. S-zonulin has also been related to a high intake of energy and proteins [7, 9, 14]. In the current study, the s-zonulin measurements were associated with the intake of proteins, fats, energy and NNS and with gut microbial dysbiosis. Specific peptides in the diet or certain bacteria in the microbiome might stimulate zonulin secretion. NNS have been associated with metabolic disorders in observational studies [27]. That NNS may impact the intestinal permeability through alterations in the gut microbiota is a hypothesis that could be explored further [28].

To explore possible explanations for the changes in s-zonulin, we analysed associations between the changes in s-zonulin after the two weight-loss interventions and the changes in the explanatory variables. After the conservative weight-loss period, the reduction in s-zonulin was associated with improvements in diarrhoea, markers of glucose tolerance and fatty liver disease (gamma-GT). Fatty liver disease is common in individuals with morbid obesity [29] and associated with impaired glucose tolerance and impaired intestinal permeability [13]. The reduction in s-zonulin during the conservative weight loss intervention might be related to amelioration of fatty liver disease [13]. The findings indicate that s-zonulin is associated with diarrhoea and IBS in subjects with morbid obesity. This contrasts to two studies in individuals with lower BMI, which did not find significant associations between s-zonulin levels and gastrointestinal symptoms and disorders [3, 30].

Many covariates were associated with s-zonulin. A post-hoc analysis with inclusion of all the significant covariates was performed to search for independent predictors of s-zonulin during the whole weight-loss period. In the final post-hoc model, four factors remained significantly associated with serum zonulin: S-glucose, CRP, protein intake and NNS intake. Biological links between serum zonulin and glucose tolerance, low-grade inflammation, proteins and NNS in the diet appear probable.

The conservative weight loss intervention used in this study led to an improvement in low-grade inflammation and glucose intolerance [17]. A regulating protein in the zonulin family might be a mediator between the diet and biomarkers. As a zonulin antagonist is available, it is possible to design studies that explore causal relations between s-zonulin and other biomarkers [2, 12]. This and previous studies indicate that individuals with morbid obesity could be a suitable group to include in an intervention trial [13].

A large reduction in zonulin was seen after the conservative weight loss intervention, at a time point when the BMI was reduced with only 3.4 kg/m^2^. In a previous study from the same cohort, the inflammatory biomarker neopterin, related to cardiovascular disease, showed a comparable pattern [31].. The weight loss after a conservative intervention is usually much smaller than the weight loss after bariatric surgery, but conservative weight-loss interventions do nevertheless have significant effects on morbidity and mortality [32]. The primary purpose of morbid obesity treatment is a reduction of the present and the future comorbidity burden, and the potential effect on BMI is only one of several factors that should be considered when treatment decisions for morbid obesity are made. The current study indicates that individuals with morbid obesity can reduce the paracellular permeability both through conservative and surgical health interventions.

It has been discussed whether the ELISA measurements of s-zonulin performed by Immundiagnostik AG measure the originally described zonulin pre-haptoglobin 2. The method might measure another protein e.g. properdin, with structural similarities to zonulin [12, 33, 34]. The current study adds to the evidence that the protein measured by Immundiagnostik ELISA is associated with the diet, gut microbial dysbiosis, diarrhoea and several biomarkers of metabolic disease. If the protein is properdin [34] and not the originally described 47 kDa pre-haptoglobin 2 [2] properdin might be an interesting biomarker and therapeutic target.

### Strengths and limitations

The strengths of this study include a prospective design with the examination of s-zonulin and a wide range of explanatory variables (diet, bowel symptoms and biomarkers) before and after conservative and surgical weight-loss interventions in individuals with morbid obesity. The explanatory variables were measured with validated instruments. Both the conservative weight loss program and the surgical procedure were effective with a weight loss of approximately ten and 27 k respectively. The study also had limitations: The diet and the microbiome were not measured at the second study visit and the waist-hip-ratio was only measured in a subset of the participants at the second and third visits. Gamma GT was the only liver function test and no radiological examination of the liver was performed. Most of the patients in our study had normal HbA1c, and our results cannot be extrapolated to subjects with diabetes mellitus. A test with a better specification of the gut microbiome would have been preferable. The sample size was limited and there were large variations in SDs for some key variables. As mentioned, s-zonulin was measured with the Immundiagnostik AG ELISA that has been problematised [34]. There were some dropouts from the study, but the missing data were missing at random, and mixed models were therefore considered as an appropriate statistical approach to handle the data set.

## Conclusions

S-zonulin, a serum biomarker of impaired intestinal permeability, was elevated above the reference value in individuals with morbid obesity. Out of several variables, p-glucose, CRP, and the dietary intake of protein and NNS were independently associated with s-zonulin. S-zonulin was markedly reduced after the conservative weight loss program, and further reduced after bariatric surgery. The reductions in s-zonulin in the whole period and after the conservative weight loss intervention were associated with improvements in diarrhoea, glucose intolerance and gamma-GT (a marker of liver disease), but not with the reduction in body mass index. The normalisation of s-zonulin may be related to the alleviation of the comorbid conditions.

## Data Availability

Case report forms on paper were used for the collection of the clinical data and are all safely stored. The data files are stored by Innlandet Hospital Trust, Brumunddal, Norway, on a server with security according to the rules given by the Norwegian Data Protection Authority, P.O. Box 8177 Dep. NO-0034 Oslo, Norway. The data are available on request to the corresponding author.

## Abbreviations

BMI: Body mass index
CI: Confidence interval
CRP: C-reactive protein
ELISA: Enzyme linked immunosorbent assay
Gamma GT: Gamma-glutamyl transferase
GSRS: Gastrointestinal symptom rating scale
NNS: Non-nutritive sweeteners
SD: Standard deviation
